# Dietary Protease Supplementation Improved Growth Performance and Nutrients Digestion via Modulating Intestine Barrier, Immunological Response, and Microbiota Composition in Weaned Piglets

**DOI:** 10.3390/antiox13070816

**Published:** 2024-07-08

**Authors:** Tao Liu, Wen Ma, Jun Wang, Yulong Wei, Yibo Wang, Zheng Luo, Ying Zhang, Xiangfang Zeng, Wutai Guan, Dan Shao, Fang Chen

**Affiliations:** 1 State Key Laboratory of Swine and Poultry Breeding Industry, College of Animal Science, South China Agricultural University, Guangzhou 510642, China; 202320156636@mail.scut.edu.cn (T.L.); 18331056296@163.com (W.M.); wangjun19992022@163.com (J.W.); a972392116@gmail.com (Y.W.); wangyibo2022@yeah.net (Y.W.); leo.luo@kemin.com (Z.L.); johnny.zhang@kemin.com (Y.Z.); wtguan@scau.edu.cn (W.G.); 2 School of Medicine, South China University of Technology, Guangzhou 510006, China; 3 Kemin (China) Technologies Co., Ltd., Zhuhai 519040, China; 4 State Key Laboratory of Animal Nutrition, Ministry of Agriculture Feed Industry Center, China Agricultural University, Beijing 100193, China; ziyangzxf@163.com; 5 National Engineering Research Center for Breeding Swine Industry, College of Animal Science, South China Agricultural University, Guangzhou 510642, China; 6 Guangdong Provincial People’s Hospital (Guangdong Academy of Medical Sciences), Southern Medical University, Guangzhou 510080, China

**Keywords:** protease, growth performance, intestinal function, intestinal microbiota, weaned piglets

## Abstract

Despite mounting evidence for dietary protease benefits, the mechanisms beyond enhanced protein degradation are poorly understood. This study aims to thoroughly investigate the impact of protease addition on the growth performance, intestinal function, and microbial composition of weaned piglets. Ninety 28-day-old weaned pigs were randomly assigned to the following three experimental diets based on their initial body weight for a 28-day experiment: (1) control (CC), a basic diet with composite enzymes without protease; (2) negative control (NC), a diet with no enzymes; and (3) dietary protease (PR), a control diet with protease. The results show that dietary proteases significantly enhanced growth performance and boosted antioxidant capacity, increasing the total antioxidant capacity (T-AOC) levels (*p* < 0.05) while reducing malonaldehyde levels (*p* < 0.05). Additionally, protease addition reduced serum levels of inflammatory markers TNF-α, IL-1β, and IL-6 (*p* < 0.05), suppressed mRNA expression of pro-inflammatory factors in the jejunum (*p <* 0.01), and inhibited MAPK and NF-κB signaling pathways. Moreover, protease-supplemented diets improved intestinal morphology and barrier integrity, including zonula occludens protein 1(ZO-1), Occludin, and Claudin-1 (*p* < 0.05). Microbiota compositions were also significantly altered by protease addition with increased abundance of beneficial bacteria (*Lachnospiraceae_AC2044_group* and *Prevotellaceae_UCG-001*) (*p* < 0.05) and reduced harmful *Terrisporobacter* (*p* < 0.05). Further correlation analysis revealed a positive link between beneficial bacteria and growth performance and a negative association with inflammatory factors and intestinal permeability. In summary, dietary protease addition enhanced growth performance in weaned piglets, beneficial effects which were associated with improved intestinal barrier integrity, immunological response, and microbiota composition.

## 1. Introduction

Weaning stands as a pivotal phase in pig production marked by various stressors, including environmental, nutritional, psychological, and social factors [1,2,3,4]. The transition from liquid to solid diets, especially high-protein feeds designed for early growth, poses a significant stress [5]. Due to the limited digestive capacity and developmental stage of piglets’ intestines, undigested proteins in their diets can potentially promote the growth of pathogenic bacteria, thereby increasing the risk of diarrhea [1]. Notably, allergenic soybean proteins, poorly digested by gastric enzymes, potentially further contribute to immune responses and intestinal damage in young pigs [6,7]. The combination of a protein-rich diet and underdeveloped intestines exacerbates stress during weaning, necessitating a focused approach to enhance the production performance of weanling pigs.

Dietary protease, an exogenous enzyme facilitating protein degradation [8], has been widely accepted and applied for supplementation into soybean meal (SBM)-based diets, aiming to enhance nutrient utilization and growth performance across various species of animals [7,9,10]. Despite the mounting evidence supporting the benefits of dietary protease supplementation, there remains a limited understanding of the underlying mechanisms beyond its capacity to improve protein degradation, a notion that has persisted for approximately 20 years [10,11,12]. Recently, there has been a heightened focus on the significant roles played by the intestinal microbiota in promoting gut health and influencing animal growth [13,14]. These roles include reducing the incidence of infectious, inflammatory, and immune-related diseases, as well as contributing to overall metabolic processes and animal growth [13,15]. Some studies in human beings and mice have indicated that inappropriately high protein levels in the diet may lead to a disturbance in the composition of intestinal microbiota, resulting in the production of toxic metabolites that induce damage to the intestinal barrier and inflammation [16,17]. A compromised intestinal barrier allows external pathogens, bacteria, and toxins to enter circulation, exacerbating the potential impact on overall systemic stress [18,19]. These investigations have prompted the hypothesis that the addition of proteases, assisting in protein digestion to mitigate protein fermentation in the intestines of weaned piglets, may regulate gut flora balance and enhance intestinal health as an additional mechanism for their beneficial effects [20,21,22].

In the present study, we conducted a comprehensive evaluation of the positive effects of incorporating proteases on the growth performance of weaned piglets. Furthermore, we delved into the underlying mechanisms by meticulously investigating the intestinal microbiota, barrier integrity, inflammation, redox status, and immunological response. These results not only enrich our understanding of, but also provide a compelling new perspective on, the beneficial mechanisms associated with protease supplementation.

## 2. Materials and Methods

### 2.1. Experimental Design and Processing

All animal procedures were conducted in accordance with the Guidelines for the Care and Use of Laboratory Animals at South China Agricultural University (Guangzhou, China) and were approved by the Animal Ethics Committee of South China Agricultural University. (No. 20230107–2, Guangzhou, China). A total of 90 weanling pigs, ([Yorkshire × Landrace] × Duroc) with an average body weight (BW) of 11.76 kg ± 0.168 (28 days of age) were used in a 28-day experiment. Pigs were allotted in a randomized complete block design, with sex and body weight at weaning as blocking criteria. They were then randomly assigned to one of three experimental diets. Each treatment consisted of 5 pens, with 6 pigs per pen (3 males and 3 females) group-housed based on body weight and gender.

The feeding experiment lasted for four weeks, during which the piglets had free access to the feed and water. The addition of essential enzyme complexes is a commonly used feed formulation technique worldwide. We were also interested in determining whether the absence of these essential enzymes would present significant challenges. Including this negative control was essential for evaluating the effectiveness of proteases. With this in mind, we aimed to provide the industry with more meaningful data on dietary proteases by including this treatment in our study. The dietary treatment groups were: (1) control (CC): a basic diet with composite enzymes without protease; (2) negative control (NC): a diet with no enzymes; and (3) dietary protease (PR): a control diet with protease. The diets employed in this study were devoid of growth promoters or therapeutic antibiotics. A basal diet was formulated to meet or exceed the nutrient requirements recommended by the NRC (National Research Council, 2012) [23] (Table 1). The proteases were in granular form, consisting of alkaline protease from *Bacillus licheniformis* (6850 units/g), neutral protease from *Bacillus subtilis* (8120 units/g), and acidic protease from *Aspergillus niger* (1700 units/g). Each unit of these proteases liberates 1 μmol of casein per minute under specific pH and temperature conditions: alkaline protease activity at pH 10.5 and 37 °C, neutral protease activity at pH 6.0 and 37 °C, and acidic protease activity at pH 3.0 and 37 °C. The basic complex enzyme includes xylanase, β-glucanase, cellulase, α-amylase, and mannanase. The composite enzyme and protease used in the experiment were both sourced from Kemin (China) Technologies Co., Ltd., Zhuhai, China.

On the 1st and 28th days of the feeding experiment, piglets were weighed and feed consumption was recorded to calculate the average daily gain (ADG), average daily feed intake (ADFI), and feed-to-gain ratio (F/G). The calculations were performed as follows: ADG = (total weight at the end of the experiment − total weight at the beginning of the experiment)/(number of piglets × number of experimental days); ADFI = total feed intake/(number of piglets × number of experimental days); F/G = ADFI/ADG. The calculation of diarrhea incidence was as follows: Diarrhea incidence = the total number of diarrhea piglets/(number of piglets × number of days tested) × 100%.

### 2.2. Sample Collection

Fecal samples were collected from days 25 to 28 of the experiment. Immediately after defecation, fresh feces were collected from each pen (6 piglets) and placed into their respective Ziplock bags. For every 100 g of feces, 10 mL of 10% H_2_SO_4_ solution was added uniformly to fix the nitrogen content of the manure. On the 28th day of the experiment, six piglets from the CC group, six from the NC group, and six from the PR group were individually selected and placed into clean crates. Rectal stimulation was administered using a sterile swab. A minimum of 5 g of fecal samples was collected and promptly transferred into a sterile centrifuge tube for subsequent 16S rRNA sequencing analysis. A 5 mL blood sample was withdrawn from the anterior vena cava of each piglet following a 12 h fasting period. Blood was obtained via jugular venipuncture into heparinized tubes and ethylene diamine tetraacetic acid (EDTA) tubes to collect plasma and whole blood, respectively. After centrifugation at 3500× *g* for 15 min, the resulting plasma samples were promptly preserved in liquid nitrogen. Following the blood collection, euthanasia was administered to all piglets through the injection of sodium pentobarbital at a dose of 50 mg/kg body weight. Tissue samples, approximately 2 cm in length, were then gathered from the duodenum, jejunum, and ileum and preserved in 4% paraformaldehyde for subsequent morphological observations. To prepare the jejunum tissue for analysis, the chyme underwent a thorough wash with cold saline. The samples were subsequently frozen in liquid nitrogen for subsequent measurements of RNA and protein.

### 2.3. Chemical Analyses

In accordance with AOAC (2012) guidelines, both diets and fecal samples underwent analysis for crude protein (CP). The fecal samples underwent a 72 h drying process in a 65 °C oven, followed by crushing and grinding through a 1 mm screen. The apparent total tract digestibility (ATTD) of both CP and gross energy (GE) was determined using Cr_2_O_3_ as an external indicator. The determination of CP involved utilizing the copper catalyst Kjeldahl method. For measuring gross energy, an automatic adiabatic oxygen bomb calorimeter (Parr Instrument Co, Moline, IL, USA) was employed. To assess amino acid (AA) content, an amino acid analyzer (L-8900, HITACHI, Tokyo, Japan) was utilized. The ATTD of both CP and GE, along with the apparent ileal digestibility (AID) of AA, were calculated by considering the chromium concentration present in the diets and digesta. The calculation formula is presented below: Digestibility (%) = (1 − A1/A × B1/B) × 100, where A represents the nutrient concentration in the diet, A1 represents the nutrient concentration in feces or digesta, B represents the chromium concentration in the diet, and B1 represents the chromium concentration in feces or digesta.

### 2.4. Intestinal Morphology

Intestinal samples were immersed in 4% paraformaldehyde and fixed for 48 h. They underwent stepwise dehydration with ethanol, followed by clearing with xylene, and were finally embedded in paraffin. Subsequently, 5 μm sections were cut at 4 °C and stained with hematoxylin and eosin. The sections were dehydrated using 90% and 100% ethanol solutions for 10 min each, followed by clearing with xylene, and then coated with a sealing agent before being covered with coverslips. Each section was observed under a light microscope to measure villus height (VH) and crypt depth (CD) using NIS-Elements BR software 3.00 (Nikon, Tokyo, Japan).

### 2.5. Blood Profile Measurements

The plasma samples from weaned piglets were centrifuged at 3500× *g* for 10 min to collect the supernatant for enzyme-linked immunosorbent assay (ELISA) detection. The supernatant was incubated with the appropriate antibody for 1 h at 37 °C, followed by incubation with the provided color developer for 30 min and washing five times. The reaction was terminated by adding a termination solution. This procedure was conducted according to the manufacturer’s instructions. The concentrations of TNF-α, IL-1β, IL-6, IL-12, LPS, DAO, D-lactate, IgG, IgM, IgA, and blood urea nitrogen (BUN) levels in plasma were determined using the Porcine ELISA Kit purchased from ML Bio Company (Shanghai, China) following the manufacturer’s instructions.

### 2.6. Antioxidant Properties

The commercial kit from Nanjing Jiancheng Bioengineering Institute (Nanjing, China) was used to measure antioxidant indexes in serum. This was performed following the manufacturer’s instructions, which included assessing malondialdehyde (MDA), reduced glutathione (GSH), glutathione peroxidase (GSH-PX), total antioxidant capacity (T-AOC), and superoxide dismutase (SOD).

### 2.7. Western Blotting Analysis

A total of 80 mg of jejunal tissue from piglets was homogenized in 1.6 mL of RIPA lysis solution (P10013B, Beyotime, Shanghai, China) containing 1% protease inhibitor PMSF (ST507, Beyotime, Shanghai, China). The supernatant was centrifuged at 12,000× *g* for 5 min. The protein concentration in the supernatant of the jejunal homogenate was determined using a BCA protein assay kit (P0010, Beyotime, Shanghai, China). Equal quantities of protein (30 μg) were separated by 10% SDS-PAGE gels (PG112, Epizyme, Shanghai, China) and then transferred to a polyvinylidene difluoride (PVDF) membrane for 1.5 h at 110 V. The membrane was blocked with 5% skimmed milk at room temperature for 2 h. After washing with TBST buffer, the membranes were immersed in primary antibody diluent at 4 °C for 12 h. Details of the antibodies used in this study are listed in Table 2. After four washes with TBST, the membrane was incubated with the secondary antibody (511203, ZenBio, Chengdu, China) at room temperature for 1.5 h. Subsequently, the target bands were visualized using an Image Quant LAS 4000 mini system and an enhanced chemiluminescence kit (P1020, Applygen, Beijing, China). Bands were quantified as gray values and normalized for relative protein expression levels with β-actin as the internal reference using Image J software (ImagePro Plus 6.0, Rockville, MD, USA).

### 2.8. Real-Time Quantitative PCR

A total of 50 mg of jejunal tissue was placed into a 2.5 mL enzyme-free grinding tube, followed by the addition of 500 μL of lysis buffer (EZB-RN001, EZBioscience, Roseville, MN, USA). Subsequent to the lysis step, RNA was processed through the column, underwent washing procedures, and was finally eluted following the provided instructions, resulting in the extraction of RNA (EZB-RN001-plus, EZBioscience, Shanghai, China). The RNA absorbance ratio (A260/A280) of all RNA samples was measured using a spectrophotometer. The synthesis of cDNA was carried out through the RNA reverse transcription reaction using the Color Reverse Transcription Kit (A0010CGQ, EZBioscience, Roseville, MN, USA), following the manufacturer’s protocol. Real-time PCR was performed utilizing the ABI Prism 7500 sequence detection system (Applied Biosystems, Carlsbad, CA, USA) with a reaction volume of 20 μL. The PCR reaction scheme included an initial denaturation cycle at 95 °C for 2 min, followed by 40 cycles of amplification at 95 °C for 15 s and 60 °C for 30 s. Primer sequences for qRT-PCR are listed in Table 3. Gene expression profiles were normalized with β-actin, and the relative mRNA expression of the target genes was calculated using the 2^−ΔΔCt^ method.

### 2.9. 16S rRNA Sequencing

Fresh fecal samples from piglets were collected and stored at −80 °C. Bacterial DNA was isolated from the feces using the MagPure Soil DNA LQ Kit (Magen, Guangdong, China). Genomic DNA from piglet fecal samples was extracted, and the V3-V4 variable region of the 16S rRNA gene was amplified using the primers 341F (5′-CCTAYGGGGRBGCASCAG-3′) and 806R (5′-GGACTACNNGGGGTATCTAAT-3′). DNA concentration and purity were assessed using 1% agarose gel electrophoresis. Qualified DNA samples were sequenced using an Illumina NovaSeq6000 (Illumina Inc., San Diego, CA, USA), generating 250 bp paired-end reads. FLASH (v1.2.7) software was used to splice the paired-end reads. ASVs and their feature lists were derived through filtering and noise reduction using QIIME software (version 1.9.1). Species annotation of the ASVs was conducted to acquire species information. The 16S rRNA gene amplicon sequencing and analysis were performed by OE Biotechnology (Shanghai, China).

### 2.10. Statistical Analysis

Statistical analysis using one-way ANOVA was performed on all data except for microbiota data using SPSS software version 22.0 (IBM Corp., Armonk, NY, USA) to identify significant differences between the groups. Results were expressed as means ± SEM. Correlations were determined by Pearson correlation analysis using GraphPad Prism 9.0 (GraphPad Software, San Diego, CA, USA). Tukey’s post hoc test was employed to assess differences among the various groups. A *p*-value of <0.05 was considered significant, and *p* < 0.01 was considered highly significant. For the analysis of fecal microbiota data, QIIME2 software was employed to conduct alpha and beta diversity analyses. Alpha diversity, comprising Simpson, Shannon, and goods_coverage indices, was calculated to assess the microbial diversity of the samples. The unweighted Unifrac distance matrix in the R package (version 2.5.3) was utilized for unweighted Unifrac principal coordinates analysis (PCoA) to evaluate beta diversity. The linear discriminant analysis effect size (LEfSe) was applied to analyze differences in the species abundance spectrum. The tool platform provided at https://www.cloudtutu.com (accessed on 2 January 2024) was utilized for performing the Kyoto Encyclopedia of Genes and Genomes (KEGG) pathway prediction analysis and constructing the correlation analysis heatmap model.

## 3. Results

### 3.1. Dietary Protease Improved Growth Performance of Weaned Piglets

The results of growth performance and diarrhea incidence of weaned piglets are presented in Table 4. The PR group exhibited higher final body weight (BW), average daily feed intake (ADFI), and average daily gain (ADG) compared to the CC and NC groups (*p* > 0.05). Compared with the NC group without enzyme preparation, adding protease to the diet increased the daily weight gain of nursery pigs from 503 to 565 g/d. Notably, the PR group demonstrated a significantly lower feed-to-gain ratio and diarrhea rate than the CC and NC groups (*p* < 0.05). There were no significant differences in feed-to-gain ratio and diarrhea rate between the CC group and the NC group (*p* > 0.05).

### 3.2. Dietary Protease Increased the Nutrient Digestion in Weaned Piglets

As shown in Table 5, compared to the NC and CC groups, the PR group had higher digestibility of crude protein and gross energy (*p* < 0.05). For the EAA, dietary protease supplementation significantly increased the apparent digestibility of Thr, Val, Met, Phe, His, Lys, and Arg (*p* < 0.05). For the NEAA, the PR group had significantly higher apparent digestibility of Tau, Ser, and Tyr than the NC group (*p* < 0.05).

### 3.3. Dietary Protease Improved Plasma Antioxidant Levels in Weaned Piglets

**Figure 1:**
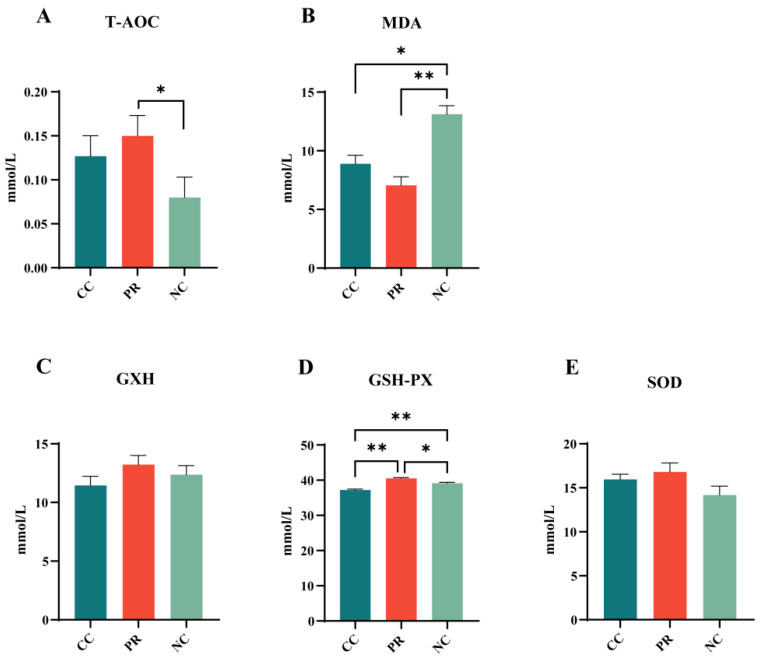
Effects of proteases on plasma antioxidant indicators in weaned piglets. (**A**–**E**) represent the changes in plasma T-AOC, MDA, GSH, GSH-PX, and SOD. All data with error bars are mean ± SEM (n = 6, 5 replicates). *, *p* < 0.05, **, *p* < 0.01.

To evaluate the impact of dietary protease on oxidative stress levels in weaned piglets, we measured plasma concentrations of T-AOC (total antioxidant capacity), MDA (malondialdehyde), SOD, GSH, and GSH-PX. These indices were used to assess the plasma antioxidant status: T-AOC reflects the overall antioxidant capacity, while MDA is a marker of lipid peroxidation. As shown in Figure 1, compared with the PR group, the level of T-AOC was reduced in the NC group (*p* < 0.01) (Figure 1A), whereas the level of MDA was significantly increased *(p* < 0.01) (Figure 1B). The CC group had a higher level of T-AOC and a lower level of MDA than the NC group (*p* < 0.05). The PR group showed significantly increased levels of GSH-Px compared to the CC group (*p* < 0.01) (Figure 1C) and a high level of GSH-Px compared to the NC group (*p* < 0.05) (Figure 1C). In addition, compared with the NC group, the level of GSH-Px was significantly reduced in the CC group (*p* < 0.01) (Figure 1D), while the SOD, GSH, and T-AOC levels did not differ significantly among the groups (*p* > 0.05) (Figure 1A,C,E).

### 3.4. Dietary Protease Reduced Blood Urea Nitrogen and Plasma Inflammation Indicators in Weaned Piglets

**Figure 2:**
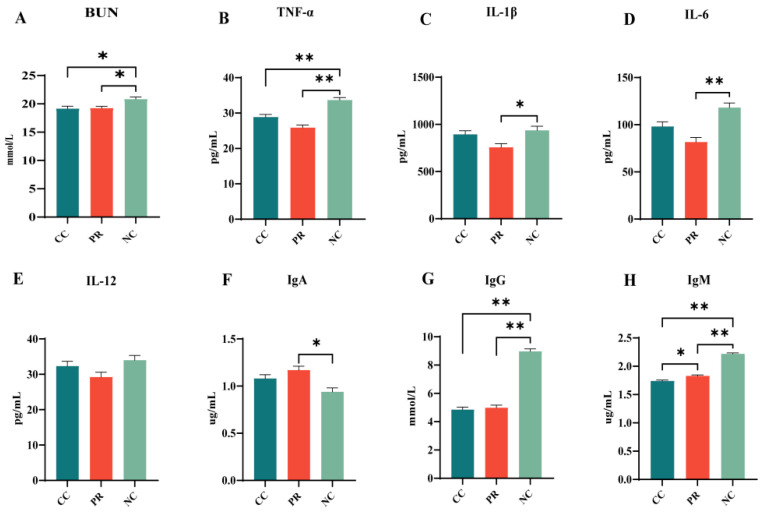
Effects of proteases on urea nitrogen, inflammation, and immunity in weaned piglets. (**A**–**E**) denote the changes in plasma levels of BUN, TNF-α, IL-1β, IL-6, and IL-12. (**F**–**H**) denote the changes in plasma levels of IgA, IgG, and IgM. All data with error bars are mean ± SEM (n = 6, 5 replicates). *, *p* < 0.05, **, *p* < 0.01.

As shown in Figure 2, besides the changes in plasma antioxidant status, the present study also investigated the effects of PR on plasma inflammatory cytokines. The NC group exhibited higher plasma concentrations of BUN, IL-1β, IL-6, and TNF-α than the CC and PR groups (*p* < 0.05) (Figure 2A,C). Conversely, the PR group showed lower levels of these cytokines than the NC group (*p* < 0.05). No significant difference in IL-12 levels was observed among the three groups (*p* > 0.05) (Figure 2D). Furthermore, the NC group had higher plasma concentrations of IgG and IgM than the other two groups (*p* < 0.01) (Figure 2F,G), whereas IgA levels were significantly different in the PR and NC groups (*p* < 0.05) (Figure 2E). These results further indicated that the PR group had a protective effect against immune stimulation, intestinal damage, and induced oxidative stress and inflammation.

### 3.5. Dietary Protease Improved Intestinal Morphology of Weaned Piglets 

**Figure 3:**
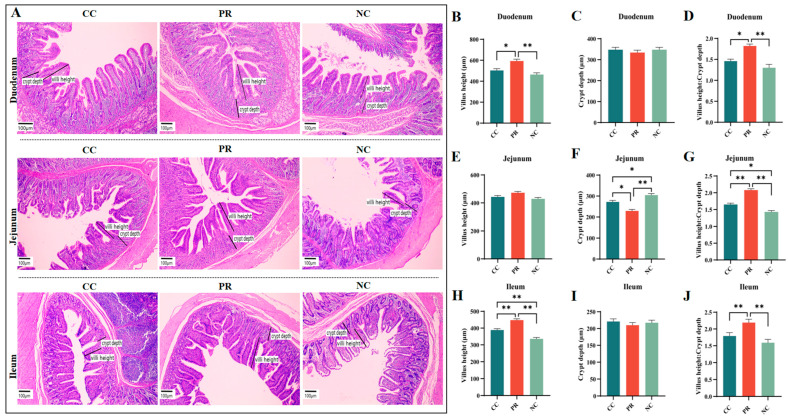
The effect of proteases on the intestinal morphology of weaned piglets. (**A**) Representative images and histomorphometry analysis of duodenum, jejunum, and ileum tissue sections from weaned piglets stained with H&E. (**B**–**D**) Villus height (VH), crypt depth (CD), and VH/CD ratio for the duodenum, respectively. (**E**–**G**) VH, CD, and VH/CD ratio for the jejunum, respectively. (**H**–**J**) VH, CD, and VH/CD ratio for the ileum, respectively. All data with error bars are mean ± SEM (n = 6, 5 replicates). *, *p* < 0.05, **, *p* < 0.01.

The intestine is essential for digestion and nutrient absorption, and its structure affects the efficiency of nutrient uptake. Figure 3A shows the differences in intestinal villus morphology among the NC, CC, and PR groups. The NC group had lower villus height and villus height/crypt depth in the duodenum and ileum, and higher crypt depth in the jejunum than the CC group (*p*  <  0.01) (Figure 3B,D,H,J). The PR group had higher villus height and villus height/crypt depth in the duodenum and ileum and lower crypt depth in the jejunum than the NC group (*p* < 0.01) (Figure 3B,D,F,H,J). The PR group also had higher villus height and villus height/crypt depth in the duodenum and ileum and higher villus height/crypt depth in the jejunum than the CC group (*p*  <  0.05). The villus height of jejunum and the crypt depth of duodenum and ileum did not differ significantly among the NC, CC, and PR groups (*p* > 0.05) (Figure 3C,E,I).

### 3.6. Dietary Protease Improved Intestinal Function and Barrier Integrity in Weaned Piglets

**Figure 4:**
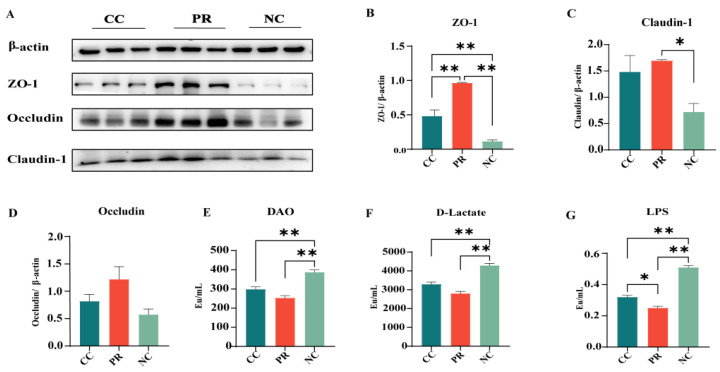
The effect of protease on the intestinal integrity of weaned piglets. (**A**–**D**) Protein levels of ZO-1, Claudin-1, and Occludin in the jejunum. (**E**–**G**) Changes in blood, DAO, D-Lactate, and LPS. All data with error bars are mean ± SEM (*n* = 6, 5 replicates). *, *p* < 0.05, **, *p* < 0.01.

Intestinal barrier dysfunction allows for the translocation of toxic substances from the intestinal lumen into the bloodstream, which can impair the growth and development of piglets. The relative protein abundance of ZO-1 and Occludin in the jejunum was significantly higher in the PR group compared to the CC and NC groups (*p* < 0.01) (Figure 4B). The PR group also showed an increased relative protein expression level of Claudin-1 compared to the NC group (*p* < 0.05) (Figure 4C). Although the expression of Occludin in the jejunum did not differ significantly among the three groups, the PC group showed a higher trend compared to the NC and CC groups (Figure 4D).

As indicators of intestinal permeability, the levels of DAO, D-Lactate, and LPS in the plasma were measured. This was carried out to further verify the efficacy of PR in enhancing intestinal barrier integrity. The results showed that the NC group had significantly higher levels of DAO, D-Lactate, and LPS than the CC and PR groups (*p* < 0.01) (Figure 4E–G). In contrast, the PR group had significantly lower levels of LPS than the PC group (*p* < 0.01) (Figure 4G), suggesting that PR can improve intestinal barrier integrity.

### 3.7. Dietary Protease Reduced Inflammation Response via Inhibiting the MAPK Signaling Pathway in Piglet Jejunum 

**Figure 5:**
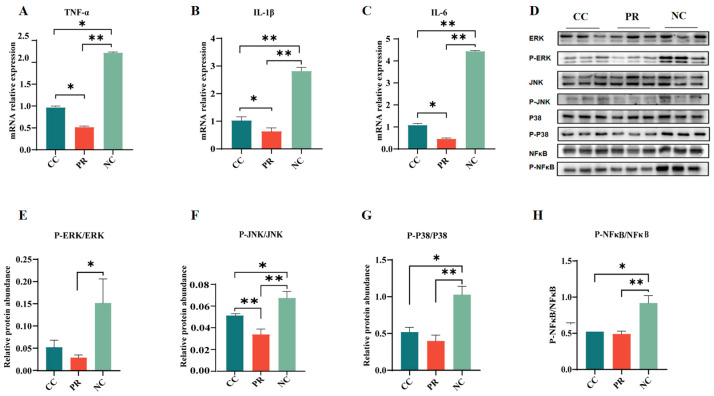
The effect of proteases on intestinal inflammation and the MAPK signaling pathway of weaned piglets. (**A**–**C**) Proinflammatory factor mRNA expression (*TNF-α*, *IL-1β*, *IL-6*) in intestinal inflammatory factors. (**D**–**H**) Western blotting was used to detect the protein levels of phosphorylated ERK (P-ERK), phosphorylated JNK (P-JNK), phosphorylated P38 (P-P38), and phosphorylated NF-κB (P-NF-κB). All data are shown as means ± SEM (n = 6, 5 replicates). *, *p* < 0.05, **, *p* < 0.01.

The expression of inflammatory factors in the intestine is pivotal for intestinal function. As shown in Figure 5, the mRNA expression of pro-inflammatory cytokines (such as TNF-α, IL-1β, and IL-6) was significantly higher in the NC group compared with the PR group (*p* < 0.01) (Figure 5A–C). Nevertheless, compared to the CC group, a significant downregulation was observed in the mRNA expression of *TNF-α*, *IL-1β*, and *IL-6* in the jejunum of the PR group (*p* < 0.05) (Figure 5A–C). The relative protein abundance of P-ERK/ERK, P-JNK/JNK, P-P38/P38, and P-NF-κB/NF-κB in the jejunum of piglets in the NC group was significantly higher than that in the CC and PR groups (*p* < 0.05) (Figure 5E–H). Moreover, the PR group showed lower mRNA expression levels of jejunal P-JNK/JNK, P-P38/P38, and P-NF-κB/NF-κB than those in the NC group (*p* < 0.01) (Figure 5E–H).

### 3.8. Dietary Protease Improved Intestinal Microbiota Composition of Weaned Piglets

**Figure 6:**
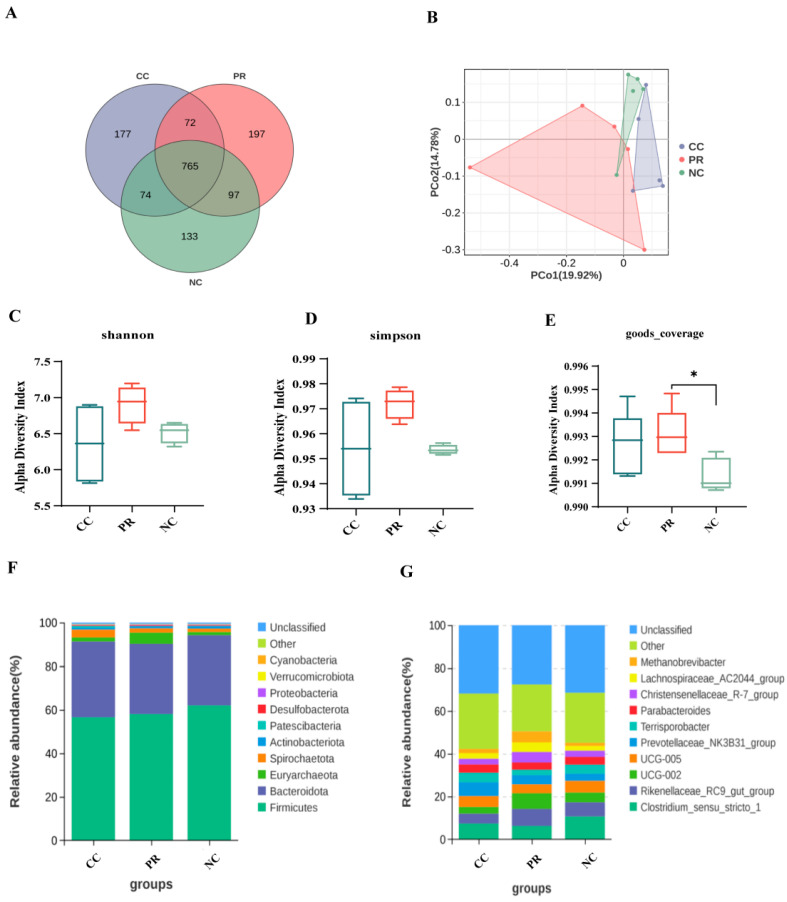
The effect of proteases on the diversity of gut microbiota in weaned piglets. (**A**) Venn diagram for the OTUs. (**B**) Beta diversity was assessed by PCoA at Bray–Curtis distances. (**C**–**E**) Alpha diversity analyses for Simpson, Shannon, and goods_coverage indices. (**F**) Relative abundance at level of microbial phylum. (**G**) Relative abundance at level of microbial genus. All data shown as means ± SEM (n = 6, 5 replicates). *, *p* < 0.05.

To assess the effect of PR on intestinal microflora, we used 16S rRNA sequencing to analyze the intestinal microbiota of piglets. As shown in Figure 6, the abundance of OTUs in the protease group was significantly increased. Specifically, the CC group, PR group, and NC group contained 177, 197, and 133 unique OTUs, respectively, and 765 OTUs were shared among the three groups (Figure 6A). In the beta diversity analysis, PCoA was performed to examine the differences in fecal microbiota composition between the PR, CC, and NC groups (Figure 6B). The alpha diversity analysis indicated no significant differences in the Shannon or Simpson indices among the three groups (*p* > 0.05) (Figure 6C,D). Nevertheless, the good coverage of the PR group was significantly higher than that of the NC group (*p* < 0.05) (Figure 6E), suggesting that protease supplementation affected the richness and diversity of the fecal microbiota. As presented in Figure 6B, there was a clear separation between the fecal microbiota communities of the three groups, indicating that PR significantly altered the fecal microbiota composition. To investigate the changes in microbial structure among three groups, we conducted a comparative analysis of the overall microbial composition at the phylum and genus levels (Figure 6F,G).

**Figure 7:**
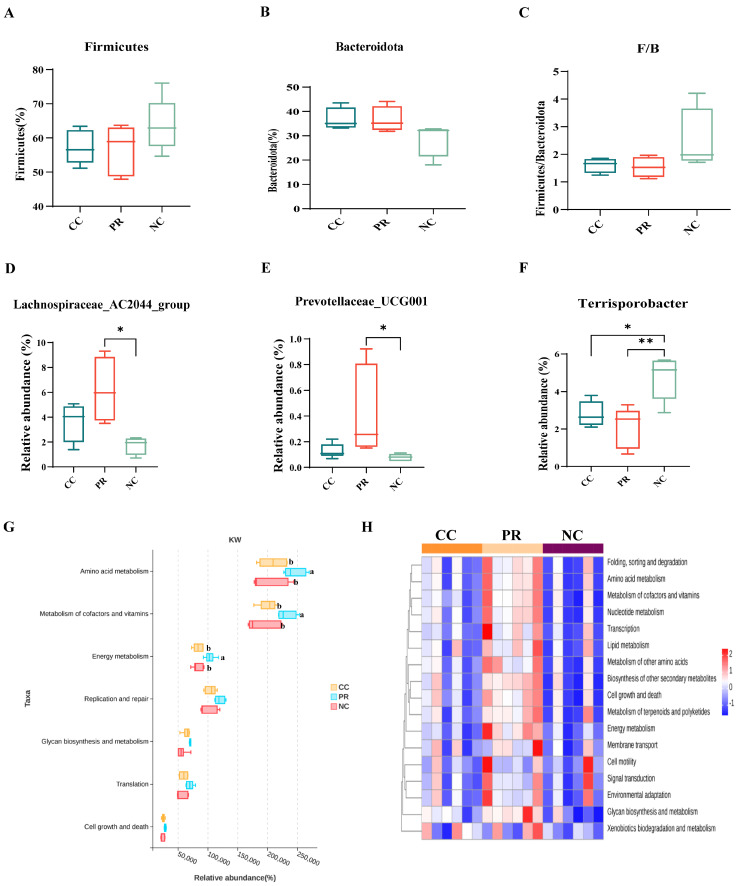
Effects of protease on intestinal flora diversity and metabolism of weaned piglets. (**A**) Relative abundance of *Firmicutes*. (**B**) Relative abundance of *Bacteroidetes*. (**C**) Ratio of *Firmicutes*/*Bacteroidetes* (F/B). (**D**–**F**) *Lachnospiraceae_AC2044_group*, *Prevotellaceae_UCG-001*, and *Terrisporobacter* between the CC group, PR group, and the NC group of relative abundance. (**G**) The metabolic pathway information of each differential gene was obtained. (**H**) The relative values are indicated by color intensity according to the legend indicated in the right corner. All data with error bars are mean ± SEM (n = 6, 5 replicates). *, *p* < 0.05, **, *p* < 0.01. ^a,b^ Different letters indicate significant differences between the 2 data.

In the NC group, there was an increase in the relative abundance of *Firmicutes* compared to the CC and PR groups (Figure 7A). Conversely, no significant differences were observed in the relative abundance of *Bacteroidota* or the F/B ratio between the groups (Figure 7B,C). Microbiological analyses revealed an increase in the beneficial bacteria *Lachnospiraceae_AC2044_group* and *Prevotellaceae_UCG-001* in the PR group compared to the PC and NC groups (*p* < 0.05) (Figure 7D,E), whereas harmful bacteria (*Terrisporobacter*) decreased (*p* < 0.05) (Figure 7F). As expected, carbohydrate metabolism was the dominant category of differential genes in the clustering base subsystem of KEGG pathways, as shown in Figure 7G, where the differential genes among the three groups were aligned with the KEGG database by the rank sum test. The amino acid metabolism, metabolism of cofactors and vitamins, and energy metabolism were significantly enriched in the PR group (*p* < 0.05) (Figure 7G). Moreover, the correlation heat map showed that the PR group had a significant positive correlation with the amino acid metabolism, metabolism of cofactors and vitamins, and energy metabolism (Figure 7H).

### 3.9. Correlation between Intestinal Microbiota, Plasma Inflammation, Intestinal Permeability, and Growth Performance of Weaned Piglets

**Figure 8:**
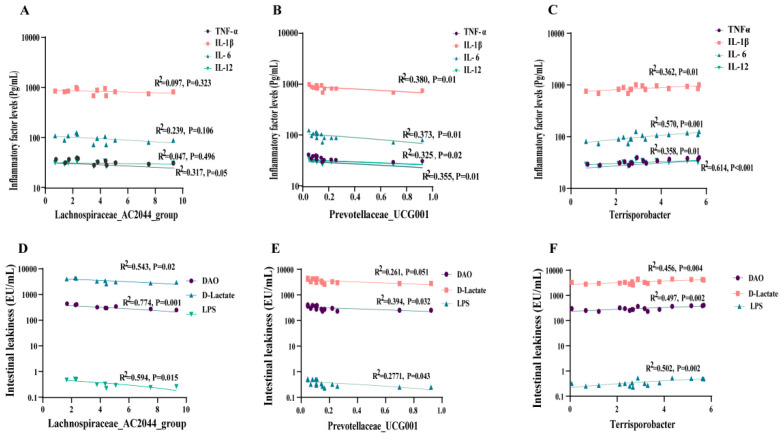
Correlation analysis of intestinal microbiota with intestinal permeability and inflammation. (**A**–**C**) Linear correlation analyses were carried out to explore the correlation between levels of inflammatory factors and the abundance of *Lachnospiraceae_AC2044_group*, *Prevotellaceae_UCG-001*, and *Terrisporobacter*, respectively. (**D**–**F**) Linear correlation analyses were performed to examine the association between intestinal leakiness and the abundance of *Lachnospiraceae_AC2044_group*, *Prevotellaceae_UCG-001*, and *Terrisporobacter*, respectively.

To explore the potential correlation between the enhanced growth performance of weaned piglets through dietary protease supplementation and alterations in intestinal microorganisms, we analyzed the correlation between gut microbiota and plasma inflammatory factors, intestinal permeability, and growth performance. The regression analysis results indicated a significant negative correlation between piglet plasma TNF-α levels (R^2^ = 0.317, *p* = 0.05) and the *Lachnospiraceae_AC2044_group*, as illustrated in Figure 8A. Additionally, *Prevotellaceae_UCG-001* exhibited negative linear correlations with TNF-α (R^2^ = 0.355, *p* = 0.01), IL-1β (R^2^ = 0.380, *p* = 0.01), IL-6 (R^2^ = 0.373, *p* = 0.01), and IL-12 (R^2^ = 0.325, *p* = 0.02), as depicted in (Figure 8B). Conversely, harmful bacteria (*Terrisporobacter*) displayed a significant positive correlation with TNF-α (R^2^ = 0.614, *p* < 0.001), IL-1β (R^2^ = 0.362, *p* = 0.01), IL-6 (R^2^ = 0.570, *p* = 0.001), and IL-12 (R^2^ = 0.358, *p* = 0.01) (Figure 8C). Further, the results revealed a negative linear correlation between beneficial bacteria (*Lachnospiraceae_AC2044_group*, *Prevotellaceae_UCG-001*) and the concentrations of DAO, D-Lactate, and LPS (Figure 8D,E). In contrast, there was a significant positive correlation observed between harmful bacteria (*Terrisporobacter*) and the levels of DAO (R^2^ = 0.456, *p* = 0.004), D-Lactate (R^2^ = 0.497, *p* = 0.002), and LPS (R^2^ = 0.502, *p* = 0.002) (Figure 8F).

**Figure 9:**
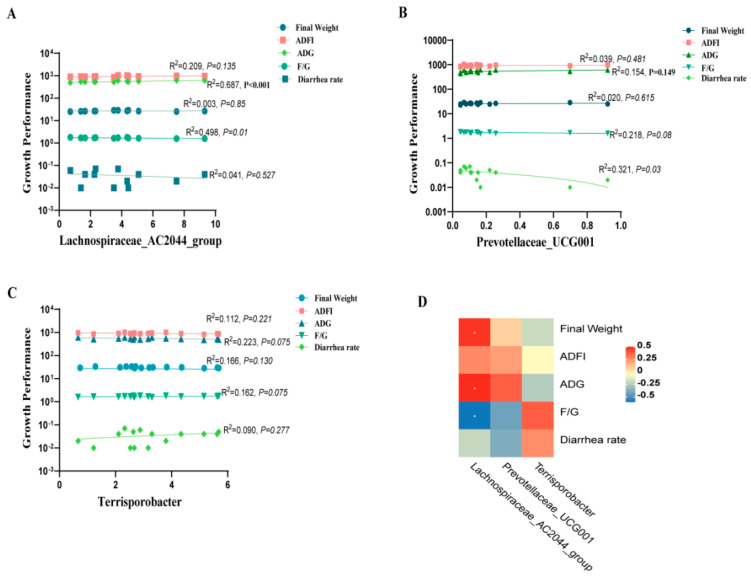
Correlation analysis of intestinal microbiota with the growth performance of weaned piglets. (**A**–**C**) Sequential linear correlation analyses were conducted to assess the relationship between growth performance and the abundance of *Lachnospiraceae_AC2044_group*, *Prevotellaceae_UCG-001*, and *Terrisporobacter*, respectively. (**D**) Correlation analysis of intestinal microbiota and growth performance. *, *p* < 0.05.

The correlation analysis revealed highly significant positive relationships between the *Lachnospiraceae_AC2044_group* and ADG (R^2^ = 0.687, *p <* 0.001), accompanied by a reduction in F/G (R^2^ = 0.498, *p* = 0.01) ratios (Figure 9A). The *Prevotellaceae_UCG-001* exhibited a significant negative correlation with diarrhea rates (R^2^ = 0.321, *p* = 0.03), as depicted in Figure 9B. In contrast, the presence of the harmful bacterium *Terrisporobacter* exhibited no significant correlation with growth performance (*p* > 0.05) (Figure 9C). Subsequently, the heatmap revealed a significant positive correlation between beneficial bacteria (*Lachnospiraceae_AC2044_group*, *Prevotellaceae_UCG-001*) and growth performance metrics (final weight, ADFI, ADG) (Figure 9D). Conversely, there was a negative correlation observed between harmful bacteria (*Terrisporobacter*) (Figure 9D).

## 4. Discussion

Weaning stress has the potential to induce intestinal inflammation, oxidative stress, and dysbiosis, ultimately leading to reduced nutrient absorption and decreased growth performance [24,25,26,27]. It has been well established that protease enzyme addition improves the growth performance of animals through enhanced protein digestion as well as neutralizing anti-nutritional components [28,29]. In the current study, we also observed improved growth performance and nutrient digestibility, particularly for crude protein and amino acids, in piglets fed with protease. This is consistent with previous investigations, further confirming its positive effects [29,30].

Weaning stress not only induces intestinal inflammation, but also serves as a primary trigger for an elevated inflammatory response [31], leading to diarrhea, reduced growth, enhanced mortality, and significant economic losses in animal husbandry [5]. Soybean meal (SBM), a prominent amino acid (AA) source in swine diets, poses constraints in weaned pig diets due to allergenic proteins, elevating the risk of allergies with the consumption of soybean products [32,33]. In this experiment, dietary protease enzymes significantly reduced inflammatory markers (TNF-α, IL-1β, IL-6, IL-12) and BUN levels in piglet serum compared to the negative control (NC) group. This benefit might be attributed to the fact that proteases enhance protein digestibility and diminish soy antigen levels in piglets, as evidenced by previous studies [7]. In biological systems, inflammation and oxidative stress are commonly concurrent phenomena that mutually reinforce cellular damage and dysfunction [34]. Consistently, our results also demonstrated enhanced antioxidant capacity in the serum of piglets supplemented with protease, as evidenced by reductions in MDA levels and increases in SOD, T-AOC, GSH, and GSH-Px levels. Further analysis focusing on the intestine suggested that incorporating enzyme preparations into the diet could also efficiently manage intestinal redox status and inflammation response. The NF-κB pathway regulates genes associated with immune and inflammatory processes, and its activation triggers the release of pro-inflammatory cytokines, resulting in tissue damage eventually [35]. Furthermore, the activation of the MAPK cascade, including P38, JNK, and ERK, plays crucial role in mediating the inflammatory signal transduction pathways induced by various stimuli [36]. We notably found that protease supplementation reduced the activation of the JNK, ERK, P38, and NF-κB signaling pathways in the intestine and reduced the proinflammatory factor of mRNA expression of *TNF-α*, *IL-1β*, and *IL-6* in the jejunum of piglets, potentially elucidating a molecular mechanism for enhanced intestinal health and nutrient digestion.

The gut barrier function is vital for the intestinal immune balance, and epithelial injury induces aberrant inflammation owing to the penetration of bacterial antigens into the lamina propria [37]. Impaired intestinal barrier function or increased intestinal permeability lead to the translocation of LPS and antigens into subepithelial tissues, which may enhance the uptake of intestinal antigens and lead to mucosal oxidative stress and systemic inflammation [38,39,40]. The VH:CD ratio can be used as an indicator to evaluate the nutrient digestion and absorption capacity in the small intestine [41]. The present study showed that weaned pigs fed the PR diet had higher VH and VH/CD ratios in the duodenum and ileum and lower CD in the jejunum compared to the NC group. These findings are consistent with those reported in previous studies [9]. Weaned piglets fed with protease supplementation in current study demonstrated noteworthy enhancements in duodenal, jejunal, and ileal morphometric indices (VH and VH/CD ratios), along with increased expression of jejunal tight junction proteins (TJPs) including Occludin, Claudin-1, and ZO-1, which are the main components of the intestinal barrier proteins [42]. Serum D-lactate and DAO are potential biomarkers of intestinal barrier function and permeability in the gastrointestinal tract [43]. Consistently, these intestinal permeability indicators were also observed, with a significant reduction in the serum of piglet from protease treatment group, further implying more strengthened intestinal integrity and barriers. The presence of undigested proteins, including soybean globulin and other antigenic proteins, can cause irritation to distinct regions of a piglet’s intestinal mucosa, which may contribute to a degree of intestinal damage and induce allergic reactions within the intestinal mucosa [32,33,44,45]. Given the detrimental impact of soy protein on the integrity of the small intestine in weaned piglets, the incorporation of protease is expected to facilitate the digestion of allergenic soy proteins [46,47]. In this study, the protease significantly mitigated intestinal damage, promoted intestinal development, improved intestinal barrier function, and decreased blood antibody levels (IgG, IgM) in piglets.

The intestinal microbiota is essential for maintaining intestinal function and overall host health, contributing to resistance against pathogens and immune stress [48,49,50]. Its regulation is influenced by numerous factors, with dietary ingredients, in particular, playing a significant role [51,52,53]. In the current study, protease supplementation significantly influenced both the composition and the function of microbial communities, as evidenced by increased microbial diversity and richness of the intestinal tract in weaned piglets. *Firmicutes* and *Bacteroidetes* were the dominant phyla in the cecal microbiota of piglets, comprising more than 80% of the bacterial community [54,55]. Previous research has suggested that F/B may be associated with the metabolic and health status of the host. A high F/B ratio has been reported to be related to diseases such as obesity, metabolic syndrome, and inflammatory bowel disease in some studies [56,57,58]. Consistent with previous studies, our results also demonstrated that the protease diet reduced the Firmicutes/Bacteroidetes (F/B) ratio at the phylum level. *Lachnospiraceae*, one of the dominant families in the *Clostridiales* order, contributes to the maintenance of gut health [59]. We found that dietary supplementation with protease increased the abundance of beneficial bacteria (*Lachnospiraceae_AC2044_group*, *Prevotellaceae_UCG-001*) and decreased the abundance of harmful bacteria (*Terrisporobacter*). The reason for this result might be the action of proteases altering nutrient digestibility in the foregut segment, leading to subsequent changes in fermentable nutrients in the hindgut and ultimately affecting the microbial structure. Further microbial function prediction analysis found that adding the protease significantly promoted metabolism, especially amino acid metabolism, followed by vitamins and cofactors and energy metabolism. It has been further confirmed that supplementing the diet with protease impacts the microbial structure and enhances the breakdown of dietary proteins. This, in turn, promotes the digestion, utilization, and metabolism of amino acids, ultimately increasing energy metabolism [12,29,30,60].

Modifications to the composition of intestinal microorganisms have a direct influence on the growth performance of weaned piglets [14]. This exploration aimed to understand whether changes in microorganisms could potentially be a mechanism contributing to the advantages observed with protease supplementation. To ascertain whether the alterations in the performance of weaned piglets are attributable to the intestinal microorganism structure or if a causal relationship exists between the two, further investigation is warranted. We further conducted a correlation analysis to examine the relationship between intestinal microorganisms and key factors such as growth performance, intestinal barrier function, and inflammatory responses. The results indicated that *Lachnospiraceae_AC2044_group* and *Prevotellaceae_UCG-001* were positively associated with growth performance and negatively correlated with indicators of inflammation and intestinal leakage, whereas *Terrisporobacter* showed the opposite effect. This significantly demonstrates that adding protease to the diet improves the gut microbial composition of weaned piglets, reduces inflammatory markers, and improves growth performance. Obviously, changes in the intestinal microbial structure of weaned piglets have a great contribution to improving the production performance of weaned piglets.

## 5. Conclusions

This study further confirms the beneficial impact of supplementing proteases on the growth performance of weaned piglets and elucidates a novel mechanism by which improvements in intestinal microbiota composition lead to reductions in oxidative stress and inflammation as well as enhancement of intestinal barrier integrity, potentially contributing to the beneficial effects of protease supplementation. These results shed light on the benefits of protease and provide a theoretical basis for its application in piglet feed.

## Figures and Tables

**Table 1:** Basal diet composition (as fed basis).

Ingredients	Content, %	Nutrition Composition ^3^	Content
Corn	27.8	DE, kcal/kg	3.45
Soybean meal	12	ME, kcal/kg	3.21
Broken rice	20	NE, kcal/kg	2.53
Five-sugar peptide ^1^	2.5	CP, %	18.48
Flour	7.5	Lys, %	1.35
Soybean oil	1	Met, %	0.50
Whey powder	2.5	Met + Cys, %	0.79
Glucose	2.5	Thr, %	0.89
Fermented soybean meal	5	Ash, %	5.24
Extruded soybean	5	Ca, %	0.78
Fish meal	4	TP, %	0.59
White sugar	5	EP, %	0.43
Mold inhibitor	0.2	EE, %	4.08
Premix ^2^	5	Salt, %	0.71
Total	100	Cu, ppm	59.10
		Fe, ppm	172.00
		Zn, ppm	1500.00
		Mn, ppm	36.07

^1^ Emulsified fat powder, used as a nursery material for suckling pigs. ^2^ The premix provided the following per kg of diets: vitamin A 10,000 IU, vitamin D_3_ 2100 IU, vitamin E45 IU, vitamin K_3_ 2.0 mg, thiamine 3.0 mg, riboflavin 5.0 mg, vitamin B_6_ 1.8 mg, vitamin B_12_ 0.03 mg, choline chloride 1000 mg, nicotinic acid 25.0 mg, pantothenic acid 15.0 mg, biotin 0.08 mg, folic acid 1.0 mg, I 0.10 mg, Se 0.20 mg. ^3^ Nutrient levels were calculated.

**Table 2:** Information on the primary and secondary antibodies used in the Western blot.

Protein	Product Name	Code No.	Company
P38	p38 MAPK Monoclonal antibody	8690s	Proteintech (1:3000, 8690S, Cell Signaling Technology, Boston, MA, USA)
P-P38	Phospho-p38 MAPK (Thr180/Tyr182) (3D7) Rabbit mAb (Biotinylated) #4092	4511S	Cellsignaling (1:3000, 4511S, Cell Signaling Technology, Boston, MA, USA)
JNK	SAPK/JNK Antibody #9252	9252S	Cellsignaling (1:1000, 9252S, Cell Signaling Technology, Boston, MA, USA)
P-JNK	Phospho-SAPK/JNK (Thr183/Tyr185) (81E11) Rabbit mAb #4668	4668S	Cellsignaling (1:1000, 4688S, Cell Signaling Technology, Boston, MA, USA)
ERK	p44/42 MAPK (Erk1/2) Antibody #9102	9102S	Cellsignaling (1:3000, 9102S, Cell Signaling Technology, Boston, MA, USA)
P-ERK	Phospho-p44/42 MAPK (Erk1/2) (Thr202/Tyr204) Antibody #9101	9101S	Cellsignaling (1:3000, 9101S, Cell Signaling Technology, Boston, MA, USA)
NF-κB	NF-κB p65 Polyclonal antibody	10745-1-AP	Proteintech (1:1000, 10745-1-AP, Proteintech, Wuhan, China)
P-NF-κB	Phospho-NF-κB p65 (Ser536) (93H1) Rabbit mAb #3033	3033S	Cellsignaling (1:1000, 3033S, Cell Signaling Technology, Boston, MA, USA)
ZO-1	ZO-1 Polyclonal antibody	21773-1-AP	Proteintech (1:1000, 21773-1-AP, Proteintech, Wuhan, China)
Occlaudin	Occludin Polyclonal antibody	27260-1-AP	Proteintech (1:1000, 27260-1-AP, Proteintech, Wuhan, China)
Claudin-1	Claudin 1 Polyclonal antibody	ab129119	Proteintech (1:1000, ab129119, Abcam, Cambridge, UK)
secondary antibody	Goat Anti-Rabbit IgG H&L	511203	Zenbio

**Table 3:** Primers and sequences used in real-time RT-PCR.

Gene Name	Gene Accession	Primer Sequences (5′→3′)
*TNF-*α	NM_214022.1	F: GCCCTTCCACCAACGTTTTC
		R: CAAGGGCTCTTGATGGCAGA
*IL-1*β	NM_214055.1	F: ATTCAGGGACCCTACCCTCTC
		R: ATCACTTCCTTGGCGGGTTC
*IL-6*	NM_214399.1	F: ACAAAGCCACCACCCCTAAC
		R: CGTGGACGGCATCAATCTCA
*β-actin*	XM_021086047.1	F: GATCTGGCACCACACCTTCTACAAC
		R: RTCATCTTCTCACGGTTGGCTTTGG

**Table 4:** Dietary protease’s impact on growth and diarrhea in weaned piglets.

Item	CC	PR	NC	SEM	*p*-Value
**Body weight, kg**					
Day 1	11.77	11.78	11.76	0.11	0.998
Day 28	26.66	27.02	25.35	0.40	0.213
**Day 1 to 28**					
ADFI, g	947	910	881	13.51	0.131
ADG, g	552	565	503	11.77	0.072
F/G, g/g	1.72 ^a^	1.62 ^b^	1.75 ^a^	0.02	0.008
Diarrhea rate, %	4.14 ^a^	2.38 ^b^	5.12 ^a^	0.33	0.051

ADFI, average daily feed intake; ADG, average daily gain; BW, body weight; F/G, ratio of feed to gain; SEM, standard error of means. All data shown as means ± SEM (*n* = 6, 5 replicates), ^a,b^ Different letters indicate significant differences between the 2 data points. (1) Control (CC): basic diet with composite enzymes without protease; (2) negative control (NC): diet with no enzymes; (3) dietary protease (PR): control diet with protease.

**Table 5:** Dietary protease impact on weaned piglets’ nutrient digestibility.

Item	CC	PR	NC	SEM	*p*-Value
Crude protein (%)	82.00 ^a^	84.39 ^b^	78.77 ^c^	0.513	0.007
Gross energy (%)	88.50 ^a^	89.70 ^ab^	84.16 ^b^	0.216	0.002
EAA					
Thr (%)	82.23 ^ab^	86.54 ^b^	81.36 ^a^	0.930	0.018
Val (%)	81.13 ^a^	83.86 ^ab^	78.98 ^b^	0.758	0.021
Met (%)	82.44 ^b^	88.24 ^a^	77.07 ^c^	1.766	0.001
Ile (%)	83.44	84.58	81.32	0.398	0.414
Leu (%)	85.16	85.83	84.44	0.357	0.320
Phe (%)	83.17 ^a^	85.65 ^b^	82.54 ^a^	0.860	0.050
His (%)	78.36 ^a^	82.84 ^b^	77.43 ^ab^	0.232	0.048
Lys (%)	85.29 ^a^	88.52 ^b^	84.78 ^ab^	0.340	0.035
Arg (%)	89.07 ^a^	91.14 ^b^	86.49 ^b^	0.179	0.029
NEAA					
Tau (%)	87.23 ^a^	92.64 ^b^	83.06 ^c^	0.130	0.026
Asp (%)	87.08	88.29	86.74	0.366	0.201
Ser (%)	86.14 ^a^	86.78 ^a^	84.83 ^b^	0.359	0.047
Glu (%)	89.11	89.81	88.67	0.225	0.088
Gly (%)	83.17	83.43	82.07	0.304	0.149
Ala (%)	82.33	82.54	81.22	0.425	0.590
Cys (%)	81.22 ^a^	82.77 ^b^	83.03 ^c^	0.400	0.125
Tyr (%)	85.76 ^a^	84.28 ^a^	81.49 ^b^	0.682	0.004
His (%)	78.36	77.84	77.43	0.232	0.288
Pro (%)	86.88	86.71	85.85	0.256	0.227
TAA (%)	86.08	86.70	85.59	0.290	0.337

All data shown as means ± SEM (n = 6, 5 replicates). Different letters (a, b, and c) indicate a significant difference among the different groups (*p* < 0.05). (1) Control (CC): basic diet with composite enzyme without protease; (2) negative control (NC): diet with no enzymes; (3) dietary protease (PR): control diet with protease.

## Data Availability

The datasets analyzed in the current study are available from the corresponding author upon request.

## Abbreviations

CC: control; NC: negative control diet; PR: dietary protease; ADFI: average daily feed intake; ADG: average daily gain; F/G: ratio of feed to gain; ANOVA: one-way analysis of variance; LPS: lipopolysaccharide; DAO: diamine oxidase; SOD: superoxide dismutase; MDA: malonaldehyde; T-AOC: total antioxidant capacity; GSH-PX: glutathione peroxidase; GSH: glutathione; BUN: blood urea nitrogen; TNF-α: tumor necrosis factor; IL-1β: Interleukin-1β; IL-6: Interleukin-6; IL-12: Interleukin-12; MAPK: mitogen-activated protein kinase; P38: p38 MAPK; P-P38: Phospho-p38 MAPK; JNK: C-Jun N-terminal protein kinase; P-JNK: Phospho-c-Jun N-terminal protein kinase; ERK: extracellular signal-regulated kinase; P-ERK: Phospho-extracellular signal-regulated kinase; NF-κB: nuclear factor kappa-B; P-NF-κB: Phospho-nuclear factor kappa-B; ZO-1: Zonula Occludens Protein 1; VH: villi height; CD: crypt depth; PCoA: principal coordinate analysis.
